# From burden to hope: Noncommunicable diseases in India and the prospects of the gut microbiome

**DOI:** 10.1002/imo2.11

**Published:** 2024-06-26

**Authors:** Preetham Kopparam, Jhinuk Chatterjee

**Affiliations:** ^1^ Department of Biotechnology PES University Bangalore India

## Abstract

This compilation aims to explore the burden of non‐communicable diseases (NCDs) in India and the potential role of the gut microbiome as a promising avenue for intervention. It focuses on understanding the unique Indian microbiome, examining the impact of microbiome interventions on general wellbeing and NCD management and outlining future directions for microbiome research in India.

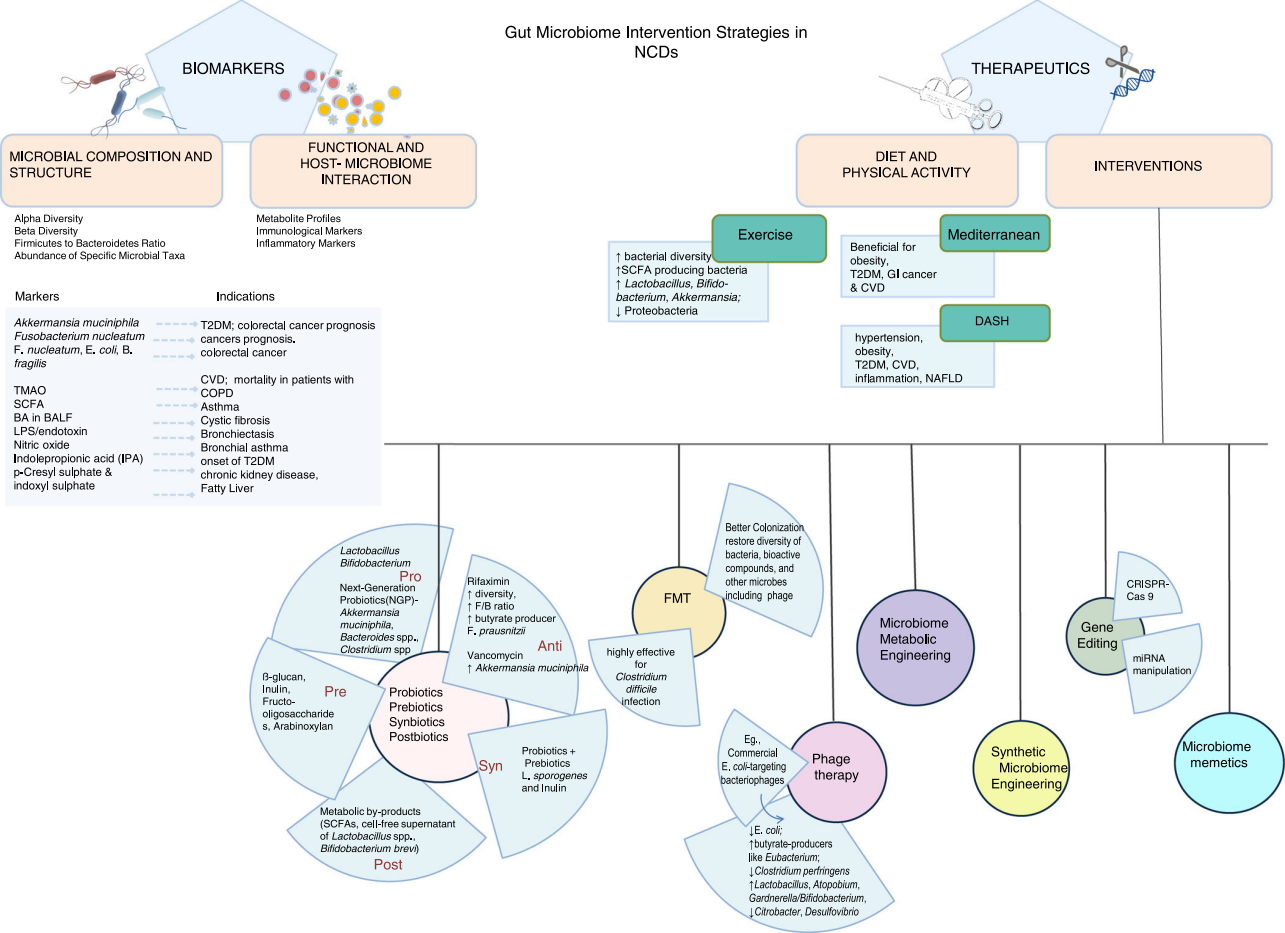

Study report by the Indian Council of Medical Research (ICMR) revealed that there is considerable increase in proportion of deaths due to noncommunicable diseases (NCDs). Four major NCDs are as follows diabetes, cardiovascular disease, cancer, and chronic respiratory disease. The prevalence of diabetes in India is considerably higher than previously estimated [1]. Chronic obstructive pulmonary disease (COPD) is the second leading cause of mortality in India [2]. India has one of the highest burdens of cardiovascular disease (CVD) [3]. In India, the incidence of cancer cases is estimated to increase from 1.46 million in 2022 to 1.57 million in 2025 [4].

Human microbiome is an aggregate of vast number of microorganisms residing at various sites in the human body. These microbes influence human physiology by contributing to metabolic functions. Various experimental findings strongly support for causal link between microbiome dysbiosis and NCDs. Changing paradigms of lifestyle, shift in dietary habits may be a contributing factor in altering human gut microbiota and its metabolic functions thereby increasing the risk of NCDs. Disturbances in microbial composition, characterized by alterations in diversity, abundance, and microbial metabolism, have been observed in various NCDs.

## 1 INDIAN MICROBIOME COMPOSITION

Composition of the Indian gut microbiome has been extensively analyzed in recent years, with a focus on identifying the core microbiome of the population. Studies have utilized multi‐omics approaches to elucidate the unique composition of Indian gut microbiome. *Prevotella* and *Megasphaera* are enriched in gut microbiome of Indians [5]. Urban populations were profiled in comparison to their rural counterparts and found *Prevotella* and *Alloprevotella* genera were more prevalent in rural areas, while *Bacteroides* and *Parabacteroides* were more prevalent in urban population [6]. Collectively, these studies provide valuable insights into genera *Prevotella*, *Bacteroides*, *Faecalibacterium*, *Roseburia*, *Eubacterium*, and *Ruminococcus* being predominant.

## 2 GUT MICROBIOME IN HUMAN METABOLISM AND HEALTH

Gut microbiota is important for maintaining host health by supporting nutrition and energy harvest. It efficiently utilizes dietary carbohydrates by producing short‐chain fatty acids (SCFAs) which exert multiple beneficial effects on energy metabolism. Studies have also associated gut microbes with total body fat content, suggesting their involvement in triglyceride storage and hepatic triglyceride production, indicating a link between gut microbiota and lipid metabolism [7]. These microorganisms also generate or modulate a repertoire of other metabolites of clinical significance like trimethylamine‐n‐oxide (TMAO) [8]. Gut microbiota‐derived products such as lipopolysaccharides (LPS), induce low‐grade inflammatory activation of resident macrophages and contribute to metabolic diseases. Further gut microbial symbionts organize into a key endocrine organ converting nutritional cues from the environment into hormone‐like signals that impact normal physiologies. These mechanistic roles of gut microbiota highlight its intricate influence on nutrient utilization, energy balance, immunological modulation, and overall metabolic homeostasis. Perturbed gut microbiota is conducive to systemic inflammation and oxidative stress leading to altered nutrient metabolism and energy balance. 1000 distinct bacterial species belong to Firmicutes and Bacteroidetes phyla are highly represented in gut and their ratio is often considered a relative estimate of intestinal microbial health.

Type 2 diabetes mellitus (T2DM) is marked by a dysbiotic microbiota characterized by lower diversity; higher ratio of Firmicutes to Bacteroidetes and reduced levels of SCFA producers. SCFAs have been extensively reported to improve glucose homeostasis [9]. Studies have reported *Bifidobacterium, Faecalibacterium*, *Akkermansia, Bacteroides*, and *Roseburia* are negatively associated with T2DM, while *Ruminococcus*, *Fusobacterium*, and *Blautia* are positively associated with T2DM [10]. Dysbiotic microbiota promote translocation of LPS, into the bloodstream, triggering chronic low‐grade inflammation impairing glucose homeostasis and promotes insulin resistance [11]. Gut microbiota influences production and secretion of various gut hormones significant for maintaining glucose homeostasis. Diabetes mellitus (DM) subjects exhibited an increased abundance of Firmicutes and *Lactobacillus* genus. Gut microbiota in T2DM consisted predominantly of Gram‐negative bacteria like *Escherichia* and *Prevotella*. Nondiabetic group consisted predominantly Gram‐positive organisms such as *Faecalibacterium, Eubacterium*, and *Bifidobacterium*. Dominant gut microbial members such as *Alloprevotella*, Rikenellaceae, *Haemophilus*, and *Ruminococcus* in Indian T2DM patients were strongly associated with both fasting blood glucose and HbA1c levels [12]. These members play a crucial role in blood glucose regulation and LPS level escalation, potentially serving as biomarkers.

According to latest studies, three classes of gut microbiome‐generated metabolites—trimethylamines (TMAs), SCFAs, and secondary bile acids are linked to CVD pathogenesis. Enterobacteriaceae, *Streptococcus* spp., *Collinsella*, *Bacteroides, Clostridium*, and Lactobacillales are considered diagnostic markers in patients with coronary artery disease [13]. In atherosclerotic plaques, there is an increased presence of bacteria from the Proteobacteria phylum (*Chryseomonas* and *Helicobacter* genera) and the Firmicutes phylum (*Anaeroglobus, Clostridium, Eubacterium, Lactobacilli* and *Roseburia*).

Gut microbiota also plays a significant role in pulmonary health and imbalances can contribute to both acute and chronic lung diseases, including COPD, asthma, tuberculosis, and lung cancer [14]. Decrease in *Akkermansia muciniphila* and *Faecalibacterium prausnitzii* are observed in patients with asthma. Abundance and diversity of gut microbiota significantly change in patients with cystic fibrosis, such as the decreases in Firmicutes, *Bifidobacterium*, *Eubacterium*, *Roseburia*, and *Alistipes* genus and the increase in *Streptococcus* spp, *Clostridium difficile*, *Escherichia coli*, *Enterococcus*, and *Veillonella*, and decrease of *Bifidobacterium* genus [15].

In cancer patients there is a prevalence of small intestinal bacterial overgrowth (SIBO). Perturbed microbiome composition promotes tumor development through broad mechanisms involving inflammation and immune response activation. *Fusobacterium nucleatum* promotes inflammation.

## 3 ROLE OF DIET IN MODELING AND REMODELING THE GUT MICROBIOME

Indian diet is rich in plant‐based foods. Promoting a healthy gut microbiome through dietary modifications, such as increased consumption of fiber‐rich foods, traditional fermented foods, and prebiotics, can positively impact the prevalence and management of NCDs [16]. Diet which is high in saturated and trans fatty acids, sugar, and low in fiber can be associated with dysbiosis. In addition to raising pro‐inflammatory bacteria that diet reduces microbial richness. Fermentation process increases beneficial bacteria, leading to production of SCFAs and reduction in plasma cholesterol.

Likewise, Mediterranean and Dietary Approaches to Stop Hypertension (DASH) diets focus on whole foods, reducing the risk of heart disease and promoting beneficial gut bacteria. Mediterranean‐ketogenic diet combines metabolic benefits with gut microbiome alterations. Additionally, low‐sodium and diverse diets influence the gut microbiota in different ways, contributing to heart health. Finally, portion control impacts the gut microbiome by regulating nutrient availability.

## 4 INTERVENTIONS TARGETING GUT MICROBIOTA DYSBIOSIS AND METABOLIC PATHWAYS

Various approaches can help restore gut microbiota homeostasis. Established therapies such as dietary interventions, prebiotics, probiotics, antibiotics, and fecal microbiota transplant are available, while novel advancements like live biotherapeutics and phage therapies offer new possibilities. Advanced strategies like microbiome mimetics, microbiome metabolic engineering, synthetic microbiome engineering, gene editing, and miRNA‐mediated microbiome modulation are actively being researched.

In diabetes management, probiotics like *Bifidobacterium, Lactobacillus*, and *Akkermansia* have positive effects on glucose metabolism [17]. Diet interventions, bacteriophages, microbiota‐targeted drugs, and postbiotics show potential in modulating gut microbiota and improving glucose homeostasis.

In CVD, probiotic administration, particularly strains like *Lactobacillus plantarum* and *Lactobacillus rhamnosus* GR‐1, has shown potential in protecting against CVDs. TMAOs, SCFAs, bile acids, phenolic compounds, and microbial peptides are key molecules with potential benefits for managing CVDs [18].

In respiratory diseases, approaches like a high‐fiber diet or short‐chain fatty acid supplementation have demonstrated benefits in reducing airway inflammation and improving lung function. Antibacterial monoclonal antibodies, bacteriophages, and predatory bacteria have demonstrated promise in preventing bacterial pneumonia [19].

Certain commensal bacteria, including *Lactobacillus* spp, *Bacillus polyfermenticus*, and *Clostridium butyricum*, have shown anticancer properties [20]. Live bacterial administration, such as *Bifidobacterium* and *Lactobacillus*, can influence anti‐inflammatory cytokines, degrade carcinogens, and produce short‐chain fatty acids, impacting cancer cell behavior.

**Figure 1:**
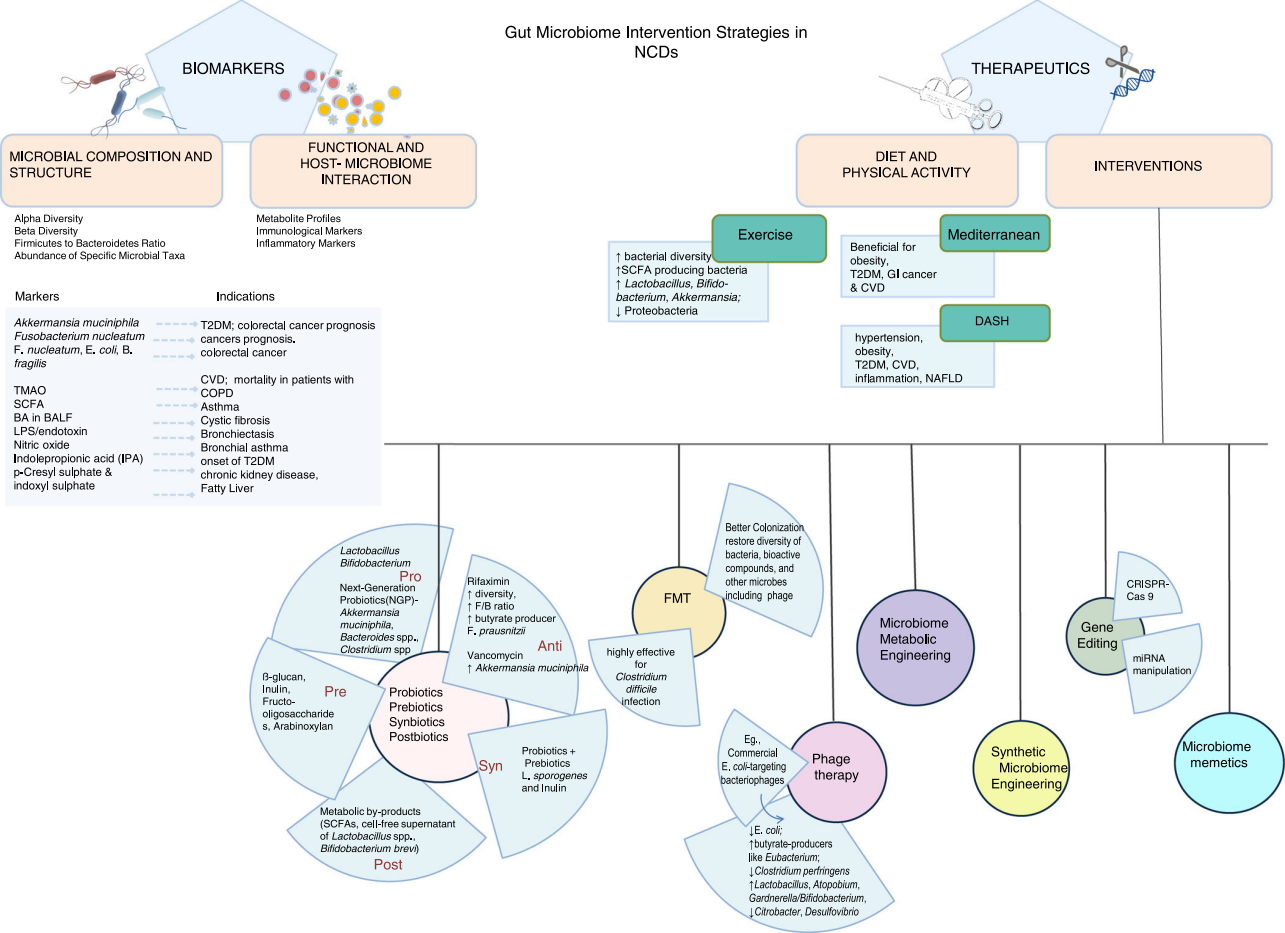
Gut microbiome intervention strategies in NCD. Microbial composition as markers with related indications are shown. Therapeutic management includes diet, exercise, and intervention strategies. Necessary details in intervention strategies include the use of pro, pre, syn, and postbiotics, FMT, phage therapy, microbiome metabolic engineering, synthetic microbiome engineering, gene editing, and microbiome memetics. BA in BALF, bile acids in bronchoalveolar lavage fluid; COPD, chronic obstructive pulmonary disease; CVD, cardiovascular diseases; FMT, fecal microbiota transplant; NAFLD, nonalcoholic fatty liver disease; NCDs, noncommunicable diseases; SCFA, short‐chain fatty acids; T2DM, type 2 diabetes mellitus; TMAO, trimethylamine N‐oxide.

Another promising approach to mitigate the burden of NCDs is to integrate these microbiome‐based interventions into the existing healthcare framework, for instance, the use of specific probiotic strains, in combination with low doses of specific drug used for treatment. This approach not only focuses on improving individual health and lowering the drugs effective concentration but also offers the potential for cost‐effective and sustainable solutions. Figure 1 summarizes intervention strategies.

## 5 FUTURE DIRECTIONS

Specific microbial taxa, along with key metabolites have been associated with these NCDs, highlighting the potential of microbiome‐based biomarkers for improved disease diagnosis and treatment. Adopting systems biology approach by integrating metagenomic and metabolomic information can offer advantages. Notably, the Indian‐ government‐funded project, “Human Microbiome Initiative of select endogamous populations of India,” demonstrates the country's progress in this field.

## AUTHOR CONTRIBUTIONS

**Preetham Kopparam**: Conceptualization; writing—original draft; methodology; writing—review and editing; visualization; investigation. **Jhinuk Chatterjee**: Conceptualization; writing—original draft; methodology; visualization; writing—review and editing; project administration; investigation.

## CONFLICT OF INTEREST STATEMENT

The authors declare no conflict of interest.

## ETHICS STATEMENT

No animals or humans were involved in this study.

## Data Availability

Data sharing is not applicable to this article as this article analyzes open‐source peer‐reviewed literature information. No new data and scripts were generated in this study.
